# A framework model using multifilter feature selection to enhance colon cancer classification

**DOI:** 10.1371/journal.pone.0249094

**Published:** 2021-04-16

**Authors:** Murad Al-Rajab, Joan Lu, Qiang Xu

**Affiliations:** School of Computing and Engineering, University of Huddersfield, Huddersfield, United Kingdom; University of Engineering & Technology, Taxila, PAKISTAN

## Abstract

Gene expression profiles can be utilized in the diagnosis of critical diseases such as cancer. The selection of biomarker genes from these profiles is significant and crucial for cancer detection. This paper presents a framework proposing a two-stage multifilter hybrid model of feature selection for colon cancer classification. Colon cancer is being extremely common nowadays among other types of cancer. There is a need to find fast and an accurate method to detect the tissues, and enhance the diagnostic process and the drug discovery. This paper reports on a study whose objective has been to improve the diagnosis of cancer of the colon through a two-stage, multifilter model of feature selection. The model described deals with feature selection using a combination of Information Gain and a Genetic Algorithm. The next stage is to filter and rank the genes identified through this method using the minimum Redundancy Maximum Relevance (mRMR) technique. The final phase is to further analyze the data using correlated machine learning algorithms. This two-stage approach, which involves the selection of genes before classification techniques are used, improves success rates for the identification of cancer cells. It is found that Decision Tree, K-Nearest Neighbor, and Naïve Bayes classifiers had showed promising accurate results using the developed hybrid framework model. It is concluded that the performance of our proposed method has achieved a higher accuracy in comparison with the existing methods reported in the literatures. This study can be used as a clue to enhance treatment and drug discovery for the colon cancer cure.

## 1. Introduction

Generally, cancer is reckoned, by the World Health Organisation (WHO), to be the second-most communal source of death in the world [1]. Colon cancer, in particular, is ranked as the third-most prevalent cancer in the United States [2]; similarly, it is ranked in third position among cancers in the UK [3] and is responsible for a large number of fatalities across the globe [4, 5].

There is short of effective medical treatment that exists for most common types of cancer [6]. One major traditional approach for detecting cancer is to use the microscopic observation of a biopsy sample that is time overwhelming, not cost effective, and sometimes ends with inaccurate results [7, 8]. Other traditional approaches are using morphological presence of tumors or parameters resulting from clinical inspections, but they may lead to imprecise results [9, 10].

As the cancer is considered to be a disease involving dynamic genome changes [10, 11], the considerable efforts have been made by researchers and technologists to explore the precise assessment and diagnose of the cancer, including the tumor prediction. Gene expression profiles using microarray data combined with computation method analysis are considered as the recent techniques and approaches toward reliable cancer features investigation and can predict more accurate results [6–10, 12, 13].

A major technological advance in classifying cancers has been the development of DNA microarray techniques, which have enabled the simultaneous measurement of a large number of genes’ expression levels [10, 14–16]. The big challenge that faces the high dimensionality of genes (features) compared to the limited sample size available [6, 9, 14, 17–24]. They might result in many redundant, noisy, and irrelevant genes (features). To overcome the high dimensionality of genes resulted from the microarray technology, there must be a way to choose a reduced subset of genes (features) from the immense number of genes, in order to produce high cancer classification accuracy, and reduce the redundant genes. Therefore, features selection becomes an important pre-requisite step for cancer classification and detection; because it reduces the redundancy and selects the most relevant genes and enhance the classification of the cells into benign (normal) and malignant (cancerous) [14, 24–26].

The current paper describes a two-stage approach to improving the successful identification of colon cancer genes. The proposed model will be composed of a pre-selection step by applying a hybrid between an Information Gain ranker and a Genetic Algorithm. Thereafter, mRMR (minimum Redundancy Maximum Relevance) filter method was applied as second stage. This mechanism is deployed to produce out a reduced subset of genes that contains an optimal subset with less noise and more relevant genes. To assess and compare the results of the proposed two stage hybrid method, a set of machine learning classification methods are used in this investigation.

The rest of the paper is structured as follows: section two presents the background and the literature review, section three presents the dataset, tools and techniques applied, while section four discusses the methodology implemented and the research approach. Section 5 renders the experimentation of the proposed method. Section six presents the results of the experiments, while section seven discusses and analyze the performance of the results. Finally, conclusion and future work are presented in section eight.

## 2. Background and literature review

In the context of the microarray technology, feature selection can be organized into three categories [14, 15, 18, 19, 27]: filter, wrapper, and embedded. In the filter method; the genes are evaluated and ranked against the class label and it does not take into considering the correlation and the interaction between the genes. It is independent from the predictor without using a learning algorithm (classifier) [28–34]. While, the wrapper method depends on adding or deleting features using the learning algorithm (classification algorithm) to assess the subset features [18, 31, 34, 35].

When comparing alternative classification algorithms, the advantage of ‘filter methods’ over ‘the wrapper method’ is that they provide a faster alternative, albeit with reduced accuracy [36, 37]. In contrast, the latter approach achieves accurate results, but with the disadvantage of being computationally slow. The embedded method, comparable to the wrapper method, applies searching algorithms for optimal feature subsets but correlated with a specific classifier construction [32, 34, 38]. The model proposed in [34] is an amalgamation of the filter and wrapper approaches and is designed to mitigate the problem of the wrapper method’s computational complexity. The approach of using such a hybrid method of classification has been used extensively in recent years to categorise cancer genes.

Other algorithms have been used for the purpose of genetic classification in the field of cancer research, featuring both machine learning algorithms and feature selection [39]. Some of these have been very successful in identifying signs of colon cancer [22, 40, 41]; the taxonomic approaches used include Genetic Algorithm (GA), Particle Swarm Optimisation (PSO), Information Gain (IG), minimum Redundancy Maximum Relevance (mRMR) (for feature selection), Support Vector Machines (SVM), Naïve Bayes (NB), Decision Trees (DT) and Genetic Programming (GP) [39, 40, 42–45].

### 2.1. Algorithms reviewed

The performance of various models in accurately classifying genes associated with colon cancers is summarised in Table 1. Among these studies, which have used algorithms based on multifilter and hybrid approaches to feature selection, four in particular [24, 46–48] have been successful in as much as they performed with an accuracy in excess of 90% in the context of colon cancers. Other approaches assessed among this sample of studies have been found to have an accuracy level of from 66% to 89%. Among these algorithms, ten of them applied a SVM as a machine learning algorithm and noted to have the highest classification accuracy [18, 46–52]. An accuracy of 90% or more was attained using GA, PSO and mRMR at a pre-selection stage.

**Table 1:** Colon cancer hybrid methods literature review for classification accuracy.

No.	Reference	Method		Accuracy
		Feature Selection	Classifier	[%]
1.	[48]	PSO+GA	SVM	91.90
2.	[46]	mRMR + PSO	SVM	90.32
3.	[47]	Genetic Algorithm (GA)	SVM	90.32
4.	[18]	CFS + Wrapper (J48)	SVM	89.03
5.	[51]	Filter (F-Score+IG) + Wrapper (SBE)	SVM	87.50
6.	[18]	CFS + Wrapper (Random Forest)	SVM	87.10
7.	[18]	CFS + Wrapper (Random Trees)	SVM	85.48
8.	[49]	mRMR	SVM	85.48
9.	[50]	mRMR+GA-SVM	SVM	85.48
10.	[52]	mRMR+GA	SVM	85.48
11.	[24]	FSBRR + MI	KNN	91.91
12.	[18]	CFS + Wrapper (Random Forest)	KNN	87.10
13.	[18]	CFS + Wrapper (J48)	KNN	85.48
14.	[18]	CFS + Wrapper (Random Trees)	KNN	82.26
15.	[53]	Genetic Algorithm (GA)	DT	88.8
16.	[48]	PSO+GA	DT	83.9
17.	[54]	GE Hybrid	DT	83.41
18.	[53]	IG	DT	77.26
19.	[55]	MF-GE	DT	76.64
20.	[48]	PSO+GA	Naïve Bayes	85.50
21.	[54]	GE Hybrid	Naïve Bayes	84.96
22.	[55]	MF-GE	Naïve Bayes	75.07
23.	[49]	mRMR	Naïve Bayes	66.13
24.	[56]	MIM+AGA	Extreme Learning Machine (ELM)	89.09
25.	[57]	Information Gain (IG) & Standard Genetic Algorithm (SGA)	Genetic Programming	85.48
26.	[54]	GE Hybrid	7-Nearest Neighbor	85.34
27.	[55]	MF-GE	7-Nearest Neighbor	68.78
28.	[54]	GE Hybrid	3-Nearest Neighbor	84.93
29.	[55]	MF-GE	3-Nearest Neighbor	77.01
30.	[54]	GE Hybrid	Random Forests	81.67
31.	[55]	MF-GE	Random Forests	74.35
32.	[58]	PCA	GA + ANN	83.33

Shutao et al. [48] isolated a subset of the top ten genes in their study in order to perform highly accurate classification, while Abdi et al. [46] used the minimum Redundancy Maximum Relevance (mRMR) technique on a pre-selected sample of 50 genes. In both cases, the size of the sample was predetermined. Mohamed et al. [47] conveyed a classification accuracy of 90.32%, using a hybrid selection approach and Support Vector Machines (SVM).

Hybrid selection was also employed by Ammu et al. [59], to ascertain information gain figures, after which a biography-based optimisation technique was used. The hybrid approach used by Chaung et al. [31], started with a genetic algorithm with a dynamic variable to select a sample of genes, which were then ordered using chi square analysis; the level of accuracy of the selection was then evaluated using SVM.

The strategy used by Dash et al. [18] was to use a combination of wrappers and filters. Feature selection was carried out using three wrappers—J48, Random Forest (RF) and Random Trees–and a sample of genes, which were assessed using the Correlation-based Feature Selection (CFS) technique. K-Nearest Neighbour (KNN) analysis and SVM were then used to measure classification accuracy. El Akadi et al. [49] initially used both mRMR and GA to study genes associated with colon cancer, verifying this approach using Naïve Bayes classifiers and SVM.

Wang et al. [60] used a two-stage hybrid method which entailed initially using a ranking procedure to obtain a sub-sample of genes. This was followed by a hierarchical grouping of the genes selected, after which an analysis was carried out using the classification algorithms C4.5, KNN, NB and SVM. The hybrid approach used by Tan et al. [61] involved a feature selection enhancement of a sample of genes using a GA; this was achieved by combining the best results from a group of feature selection methods, after which SVM were used to analyse the data. Kim and Cho [62] classified genes by employing an evolutionary neural network, while Mohamad et al. [19] made their selection of genes from microarray data, using a Cyclic-GASVM hybrid method. In a separate study, Mohamad et al. [63] used a variation of the GASVM, (referred to as “GASVM-II + GASVM”), for the gene selection process.

An alternative means of feature selection was used by Hanaa et al. [57], using a combination of GA and information gain; subsequent analysis was carried out using Genetic Programming (GP). Elyasigomari et al. [64] applied “MRMR-COA-HS”, which first used mRMR to make a selection of genes, before using a wrapper which involved an algorithm known as COA-HS and SVM for classification. Alshamlan et al. [17] also used SVM at the final stage, having carried out feature selection using both mRMR and an ABC algorithm. Shukla et al. [22] presented a two-stage selection approach composed of the combination of Spearman’s Correlation (SC) and the distributed filter FS methods. In [35] Shkula et al. had proposed another hybrid wrapper method to obtain the key gene expressions which is composed of Correlation-based Feature Selection (CFS) as the first step, followed by the TLBO algorithm as the second step. The accuracy has been ranged from 92.23% to 88.52% [35].

Table 1 had listed 32 different approaches of applying the hybrid feature selection method, 4 of these methods had achieved a better classification accuracy of 90% or above. Most of the state-of-the-art technologies found that for the colon cancer dataset, the mRMR, GA, IG, and PSO are commonly applied for the hybrid feature selection and evaluates to better results.

### 2.2. The limitations of previous studies

In the light of above, the limitations of previous studies are highlighted below.

- The most literatures reported good results when they limited the quantity of gene selection to a fixed number of genes prior to classification, thus ignoring the rest of genes which may cause an ignore to important gene.
- Many studies had claimed that reducing the number of genes will enhance the classification accuracy, but as shown in Table 1 the superlative accuracy reached 92%. Thus, there is a need to a better method or a framework model to proof the classification enhancement of the hybrid methods.
- To the superlative of the author’s knowledge, there is no previous study stated in the literature had touched the hybrid feature selection method with the approach of a two-stage multifilter hybrid selection method.

### 2.3. The objectives of investigation

The main objective of this investigation is to develop a new framework for selecting colon cancer genes, in two stages, the first comprising a multifilter hybrid stage (GA+IG) to optimize the quality data from dataset, and the second consisting of an mRMR procedure for making the final selection. The both stages will work as selection algorithms along with machine learning classifiers to predict the cases of colon cancer. These hybridizations of algorithms are proposed to obtain genes subsets with a minimal number of relevant genes, which thereafter can produce high classification accuracy that can be employed to better detect colon cancer.

To overcome the limitations mentioned in section 2.2, in this study we use three selection algorithms (GA, IG, and mRMR). This combination is different from previous two stage approached, and we tested the accuracy using four classifiers: SVM, NB, DT and KNN to ensure the investigation is conducted rigorously. The reasons to employ these algorithms are: 1) They had shown better performance than other selection algorithms in the field, and had reflected very good effectiveness in many colorectal cancer research studies [39–41]; 2) GA has the ability to manage high dimensionality datasets for the colon cancer [65–67]; 3) GA can achieve interesting results when combined with other algorithms [68]; 4) GA is easily integrated and worked in parallel with other algorithms; 5) IG had advantages in eliminating redundant genes and reducing noise [26, 69, 70].; 6) Combining the GA and IG in stage 1 of this framework model will achieve the target of generating a subset of features which are top ranked and with very good quality; 7) utilizing the mRMR in as a multifilter in stage 2 will refine the subset generated from stage 1 through another subset selection of features. These features are more correlated and relevant with the class that has the lease correlation between the features. It follows that all of these algorithms will be expected to result in very good interruptible gene expressions in order to achieve a better identification to the colorectal cancer disease.

## 3. Dataset, tools and techniques applied

The datasets that are used for the colon cancer throughout the study are described in this section; the tools used for the experimentation, and outline the selection techniques used.

### 3.1. Background of the dataset

In this paper two datasets were used. The first one was collected from Alon et al. [71], which has been used in several colon cancer research studies [18, 46–57, 59, 72]. This dataset is publicly available and is still utilized in most recent studies [22–24, 35, 57, 56, 73–77]. Moreover, to mandate the performance of the proposed model, another colorectal dataset was used. This dataset was collected from Notterman [78], which is also used in recent studies [8, 79, 80]. Both data sets are publicly available and acquired as gene expressions. Table 2 presents the details of these two datasets.

**Table 2:** Description of the datasets’ gene expression used in the study.

TYPE OF DATASET	NO. OF GENES ACROSS THE SAMPLES	CLASSIFICATION TYPE	NO. OF SAMPLES	
Alon et al. [71]	2000	Tumour	62	40
		Normal		22
Notterman [78]	7457	Tumour	36	18
		Normal		18

### 3.2. Tools utilised

The Weka machine learning environment is employed in this research https://ai.waikato.ac.nz/weka/, as the Weka resource provides a number of techniques that can be used for data validation. Two such techniques are ‘leave-one-out cross-validation’, or LOOCV, and *k*-fold cross-validation, both of which randomly classify items of data as being part of either ‘training’ or a ‘testing’ set [81, 82]. The LOOCV approach involves a ‘classifier’ being learned for all bar one of a sample and tested on that one data point [83]. The *k*-fold cross-validation technique is different in that the data are divided into an equal number of sub-samples. Each sub-sample is tested once and then used for training; this process will be repeated *k* times to make sure that all sub-samples are tested [84].

### 3.3. Feature selection techniques

Improving the accuracy of predictions by identifying certain features on the grounds of correlation statistics is known as ‘feature selection’. For a dataset D having d dimensions, feature set F can be expressed as:

$$F=\{f_{1},f_{2},\ldots ,f_{d}\},$$ (1)

where F stands for the feature set. The objective is to deduce an optimum group of features F’, where (1) F’ ⊆ F and (2) F’, since this will represent a very good rate of classification. On the other hand, the classification process is the way to present out the test accuracy of the result. It is also possible, using this technique, to assess accuracy as a function of the ratio of predicted samples to total samples.

## 4. Methodology

In this section, a description will be given of the methodology used, including the system design and the creation and use of the appropriate algorithm.

### 4.1. System design

**Fig 1:**
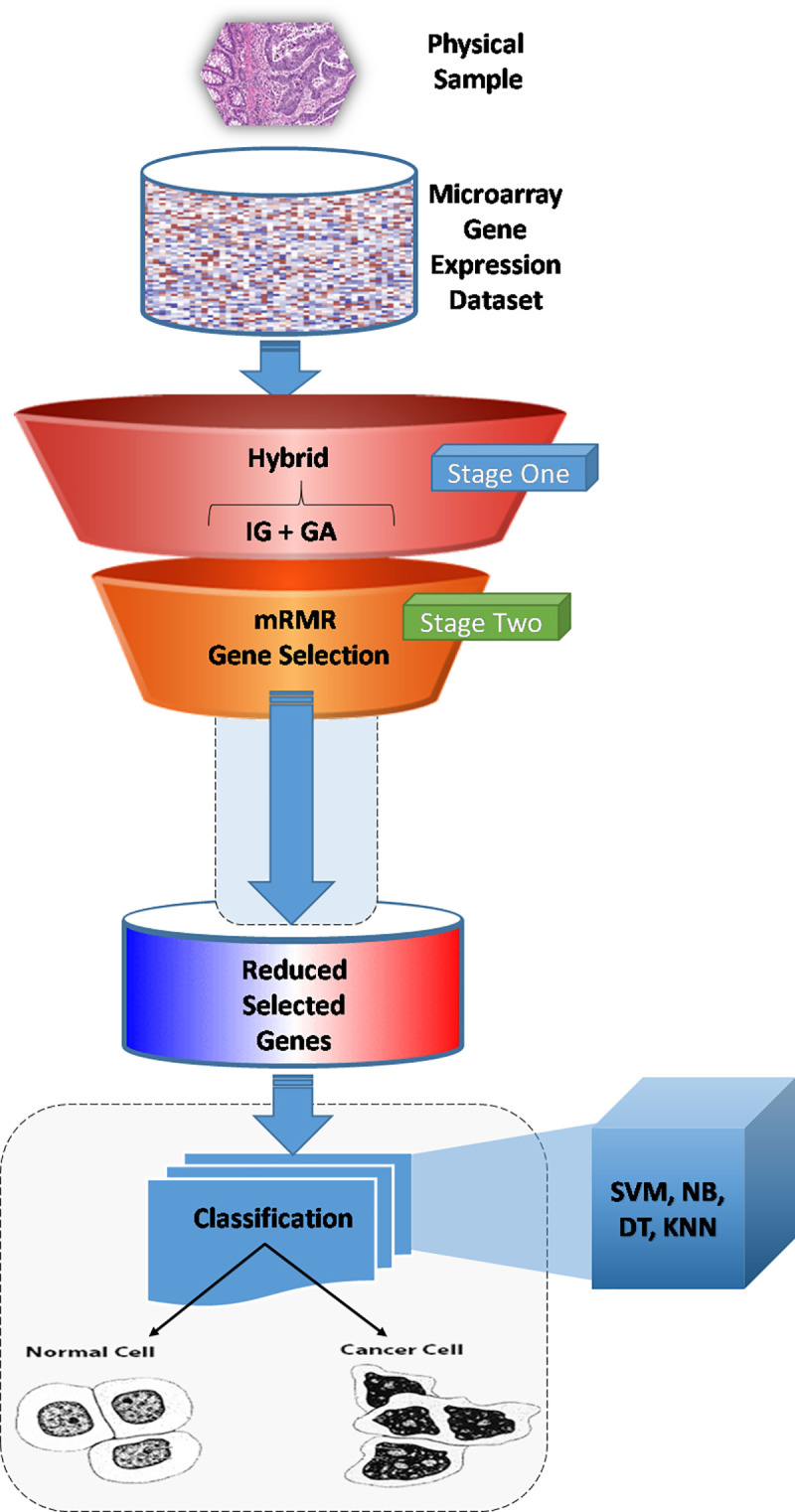
Proposed computational model framework.

The key contribution of this research is to develop an original framework for the two-stage multifilter hybrid method for colon cancer feature selection, to achieve better classification accuracy shown in Fig 1. What makes our method distinguished in comparison to those in the literatures is that we contributed to the whole colon cancer dataset (all genes), while previous studies are rarely reported, and didn’t restrict the accuracy evaluation to a particular number of top genes in the selected subset. In addition, we applied the same machine learning algorithms without any option of parameters tuning.

Although the use of hybrid models appears in the research literature, the novel aspect of the present study is that it sets out to decrease the number of genes selected and enhance the accuracy of the classification by means of a multifilter two-stage feature selection process.

The rationale for striving to improve on current selection and ranking approaches is that they rely on a one-stage process and the probability that their results contain the ‘noise’ of redundant and unrelated genes still exist. The current study tries to alleviate this problem using a two-stage, multifilter technique, which proceeds as follows:

- As a first stage, a hybrid procedure (GA+IG) is applied to the entire dataset, which both selects genes (GA) and ranks them (IG). The key idea of utilizing this hybridization is that the IG will rank the genes according to their importance, while GA is considered a well-known algorithm to find an optimal solution and easy to implement. Both algorithms will refine and reduce the dataset for stage two and for the classification thereafter.
- Then we filter out the selected features using a second stage of ranking genes (mRMR), which will remove redundant genes, reduce noise, and leave only correlated genes in the newly subset selected.

**Fig 2:**
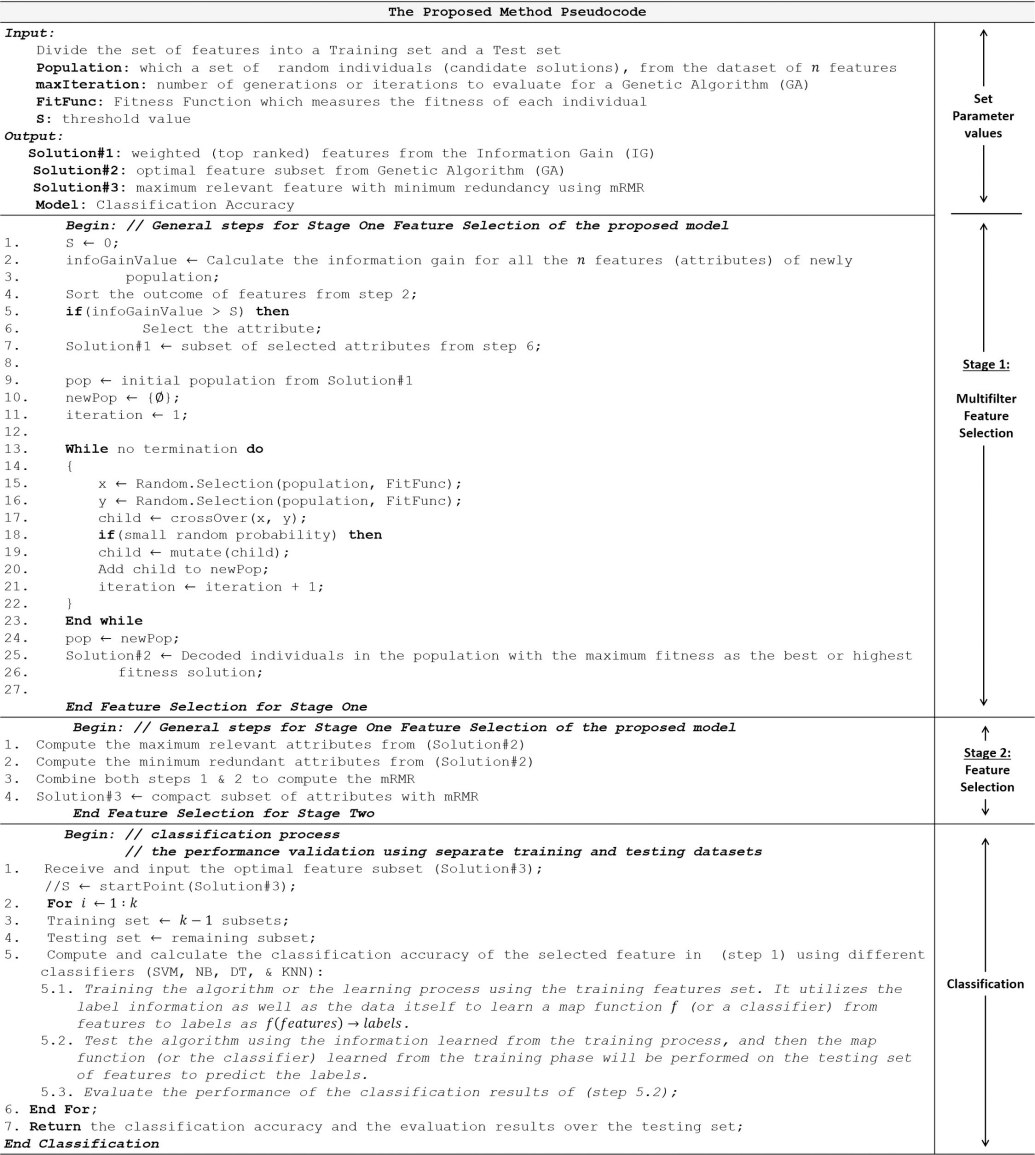
Pseudocode of the proposed model.

As rendered in Fig 2, the procedure is as follows:

- The raw dataset comprises of actual tissue samples obtained from patients suffering with colon cancer, prepared for analysis in the form of a microarray.
- The comprehensive gene expression information that is contained within the microarray is prepared in a format that enables analysis using the appropriate computer programs.
- The first phase of the analysis is to process the data to reduce the ‘noise’ in the dataset and to perform some initial categorisation, to improve the accuracy of the subsequent classification. This consists of a two-stage action:
  ○ 1. The (GA+IG) hybrid procedure
  ○ 2. Feature selection, consisting of mRMR and Stage 1 hybridisation
- Assessment of prediction accuracy, which is performed with a number of classification algorithms, such as SVM, NB, DT and K-NN. This final stage provides an evaluation of the accuracy with which a cell from a patient can be diagnosed as being cancerous or normal.

The following paragraphs outline the proposed framework model, whilst Fig 2 presents the pseudocode. To recap, the overall objective of this work is to improve the accuracy with which cells are classified as being cancerous or non-cancerous, with the approach of this work being to improve feature selection so that a better subset of genes is used for the analysis, including genes that are more closely related.

### 4.2. Definitions and descriptions

It is assumed that the dataset subjected to the initial GA process is m-dimensional and that the format of the data can be defined by the matrix (*Data*_*n*×*m*_), where *n* represents the number of data points (individuals being treated for colon cancer, in the current context) and *m* is the number of genes involved in the analysis. The process of multifilter feature selection has the objective of deriving the best possible subset of features for the analysis. Let the initial set of features, *X*, having *m* dimensions, be defined by the equation *X* = {*x*(*i*)|*i* = 1,2,3,…,*m*} where *x*(*i*) are the defined features and *m* are the genes. The feature selection process, IG, is used to derive *Y*, which is calculated as *Y* = {*y*(*i*)|*i* = 1,2,3,…,*p*} where *y*(*i*) are the selected optimal features and *p* represents the revised set of genes. The next step in the method is to rank all of the genes (features) in terms of the amount of information that is derived from including each one, with the criterion for inclusion being a positive value (i.e. an information gain threshold value of above zero). This ordering is passed out to identify the features that have the greatest influence on the classification of the genes. *Y* must be an optimal subset of *X*, so that *Y*⊂*X*, and *p*≤*m*. The features *y*(*i*)∈*Y* are then subjected to GA, to create the vector *Z* = {*Z*(*i*)|*i* = 1,2,3,…,*q*}, where *Z*(*i*) represents the new subset of features and *q* is now the number of features in the subset, although *Z*⊆*Y* and *q*≤*p*. A disadvantage of using the IG procedure is that the features are dealt with separately, so that the correlations between them may be lost. Using mRMR minimises redundancy in the process, due to its emphasis on high relevance and close correlation; in the context of the *Z* data, mRMR identifies features that are strongly relevant to the task of classification and which carry with them the least redundancy, thus deriving an original set of vectors *A* = {*A*(*i*)|*i* = 1,2,3,…,*s*}, where *A*(*i*) is the final subset of features and *s* is the number of features. In this case, *A*⊆*Z* and *s*≤*q*. In the next phase, the vectors *A* are categorised in terms of whether they refer to a tumour or normal tissue, using the binary labelling system {−1,+1}. This provides a new dataset of genes, which is defined by the equation ${\left\{(l(A_{i}),C_{i})\right\}}_{i=1}^{l}=l(D)$, with $l(A_{i})\in R^{m^{\prime }}$, where l selects *m*′<*m* features from *n* genes, and *D* represents the microarray of gene expressions.

The effect of the procedure described here is to create a situation where *F*:*A*→*C*, whereas previously *F*:*X*→*C*.

## 5. Experimentation

This section presents the data preparation, the instrumentation tools, the experiments design, and the experiment process.

### 5.1. Data preparation

An issue that needs to be overcome in gene research is that any set of data analysed will be small in relation to the total gene population. Furthermore, the global genetic dataset is characterised by ‘noise’ and redundant information [85]. Using feature filtering techniques is considered one way to address this situation which prepares the raw data into a suitable form for analysis.

A popular method to pre-process the data is to discretise it using the entropy-based discretisation method proposed by Fayyad & Irani [86]. The approach used in the present study as a means of global discretisation is one that has already been used elsewhere [10, 16, 18, 49, 87, 88]. Since the first dataset is unprocessed [71], then we discretised the original data into categorical ones to minimize and eliminate the noise. This algorithm applies an entropy minimization heuristic recursively to discretise the continuous-valued attributes. The stop of the recursive step for this algorithm depends on the minimum description length (MDL) principle [10]. However, the second dataset [78] is being processed by first removing any duplicated genes to keep only the unique ones, and then each array is being standardised into zero mean and unit variance. It is found that 860 duplicates exist, and they were removed.

When using GA, GI, mRMR and the selected classification process, certain default assumptions were made initially, namely the sample population for GA was 20 and the termination criterion was 20; similarly, the crossover probability was 0.6 and the mutation probability was 0.033, and the IG threshold was fixed at zero.

### 5.2. Instrumentation and resources used

The experiment was conducted using the Weka machine learning environment and the related library packages, with default values for all parameters [11, 89]. The computing environment used a PC with the Windows 10 operating system, a 1.8GHz Intel Core i5 processor and 8GB of installed RAM.A number of programs were used for the analysis, including Windows 7, Windows 8, Intel Core i7 and 16GB RAM, but this did not affect the output obtained.

### 5.3. Experimental design

Prior to starting the analysis, the data were separated into two sets: training and testing, in order to create an independent test set, and improve the validity and the accuracy of the classification. The experiments were using different testing models (K Fold cross-validation, LOOCV, and splitting into training and testing proportions). As the number of samples in the datasets are considered small, the 10-fold cross validation is adopted as a value for the cross validation [90]. We also adopted the testing model to divide the samples into training almost (70%) and testing about (30%). The creation of the training set enabled a validation of feature selection; the test set fulfilled a similar validation role in relation to the classification process. It is significant to note that in the proposed method to implement cross validation, we separately discretised the training set for each fold in order not to have an access to the testing data, which will result in optimistic error rates and compromise the reliability of the experiment. Thus, during the dataset training process, the test set will be unseen (hidden) to assure the validation of the results when applied to fresh data.

### 5.4. Experimental processes

Data preprocessing techniques were carried out on the datasets prior to the analysis (see Section 5.1). Features were then selected using the following two-stage approach:

- Stage 1: Discriminative scores were derived for each gene using IG, and all genes with a score of zero were eliminated from the dataset. Genes providing a large amount of information were selected, using GA, in order to optimise the dataset with informative and correlated genes.
- Stage 2: Redundancy levels were further reduced using mRMR, to maximise the efficiency of the gene selection process. The objective, here, was to reduce the number of features in the analysis to a minimum and to lower the amount of ‘noise’ in the data. mRMR was used to derive a subset of preferred genes.

**Fig 3:**
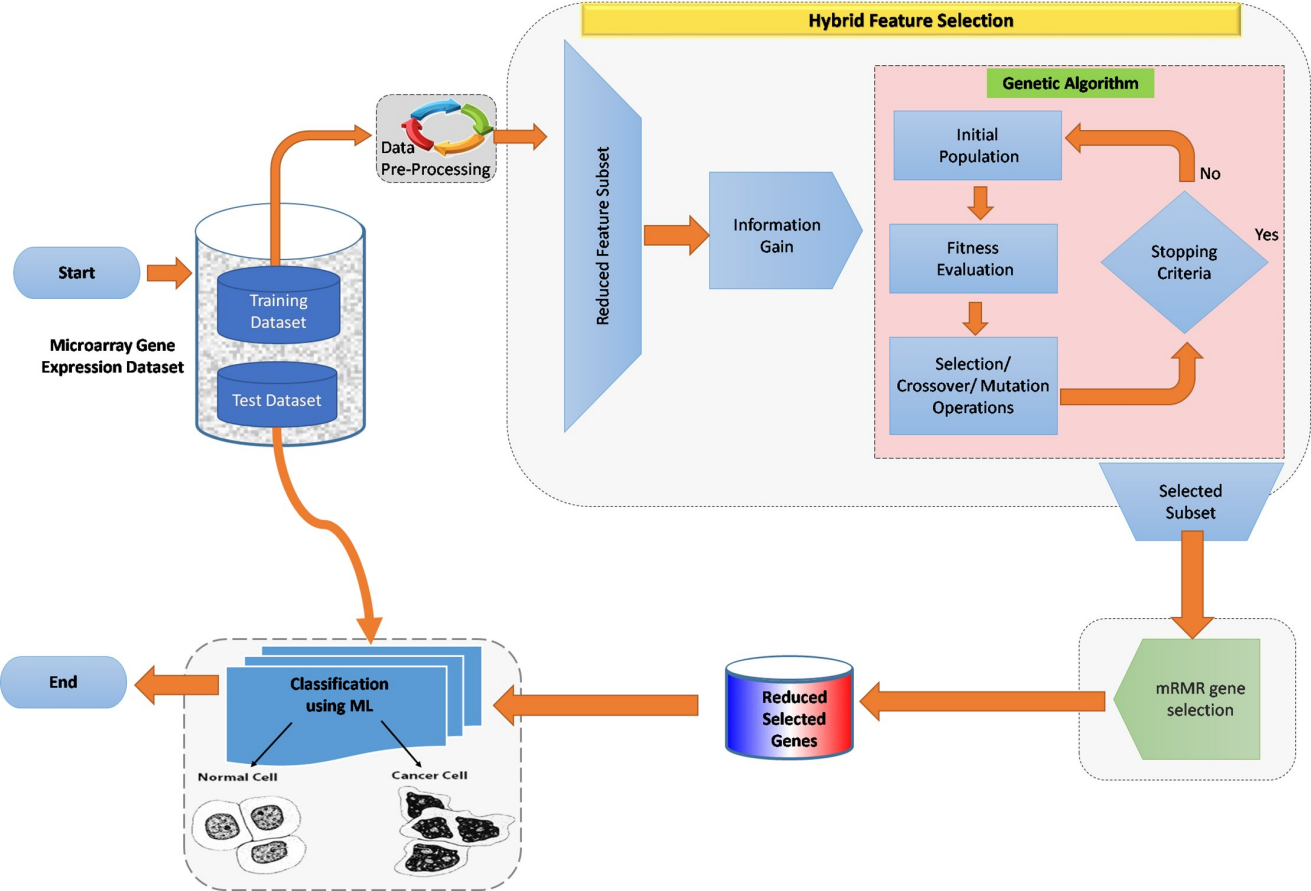
The summary of the developed multifilter 2-stage framework method.

Next, the classifications carried out were evaluated using a number of approaches–DT, the K-NN, NB and SVM–in order to identify the best and most efficient classification algorithm, and to measure the classification rate. Fig 2 shows the pseudocode used in the method described in sub-section 4.2. The first step was to rank the features by the extent of their information gain, using IG, after which subset features were searched using the GA technique. The evaluation, which dealt with each gene in turn, used a fitness function. The next step was to derive a new, improved population, by selection, crossover and mutation; this was repeated until pre-defined criteria for halting the process were achieved. mRMR was then used on the subset of genes obtained by this method, retaining only genes that had high relevance and which were closely correlated with one another. Finally, an evaluation was carried out, employing a number of algorithms, of the quality and accuracy of the classifications. A summary of the implementation of the research is provided in Fig 3.

## 6. Experimental results

As described above, a two-stage hybrid approach (IG+GA) and (mRMR) were used for the selection of features, followed by subsequent classification. The results obtained will now be presented in terms of the features selected number and of the outcome of the evaluation of the quality of the classification process.

### 6.1. Number of selected features

Table 3 shows the results of Stage 1 of the analysis–feature selection. From the initial sample population of 2,000 genes from the first dataset (Dataset 1), a subset of 68 genes was selected, based on the parameters Information Gain and Genetic Algorithm and 475 genes were selected at the same stage from the second dataset (Dataset 2).

**Table 3:** Number of selected features by the proposed method on each dataset.

	Colon Features	
	Dataset 1	Dataset 2
Full Data Set	2000	6597*
Phase 1 (IG+GA)	68	475
Phase 2 (Phase 1 + mRMR)	22	35

* this number after eliminating duplicates.

On stage 2 of the analysis mRMR is used to rank the gene population according to each gene’s level of redundancy and level of correlation with the other genes. This resulted in the creation of a subset that minimised redundancy and maximised the chosen genes’ contribution to the classification process. As Table 3 shows, a total of 22 features had been included at this stage of the process from dataset 1, with the original dataset having been reduced by almost 99%. However, 35 features had been included at this stage of the process from dataset 2, reducing the original dataset by almost 99.5%.

Table 4 illustrates that the top genes are ranked, selected, and considered to be as the key genes in the occurrence and the development of colorectal cancer. The table contains the Expressed Sequence Tag Number (EST) and Genes Expression Description. For example, some of the key features in dataset 1 are M26383, M63391, M76378, J02854, and T968730, while in dataset 2 are R36977, M77836, T96548, T64297, and M97496 as the key gene expressions based on the proposed model.

**Table 4:** Top genes ranked and selected according the proposed framework model.

Dataset 1	Dataset 2
M26383 gene 1 "Human monocyte-derived neutrophil-activating protein (MONAP) mRNA, complete cds."	R36977 yf53h07.s1 Homo sapiens cDNA clone 26045 3’ similar to SP:TF3A_XENLA P03001 TRANSCRIPTION FACTOR IIIA;
M63391 gene 1 "Human desmin gene, complete cds. "	M77836 "Human pyrroline 5-carboxylate reductase mRNA, complete cds"
M76378 gene 1 "Human cysteine-rich protein (CRP) gene, exons 5 and 6. "	T96548 "ye49f12.s1 Homo sapiens cDNA clone 121103 3’ similar to gb:X16940 ACTIN, GAMMA-ENTERIC SMOOTH MUSCLE (HUMAN);"
J02854 gene 1 "MYOSIN REGULATORY LIGHT CHAIN 2, SMOOTH MUSCLE ISOFORM (HUMAN);contains element TAR1 repetitive element;."	T64297 "yc48a10.s1 Homo sapiens cDNA clone 83898 3’ similar to gb:M10050 FATTY ACID-BINDING PROTEIN, LIVER (HUMAN);"
T96873 3’ UTR 2a 121343 HYPOTHETICAL PROTEIN IN TRPE 3’REGION (Spirochaeta aurantia)	M97496 "Homo sapiens guanylin mRNA, complete cds"
U21090 gene 1 "Human DNA polymerase delta small subunit mRNA, complete cds. "	X64559 H.sapiens mRNA for tetranectin
H40560 3’ UTR 1 175410 THIOREDOXIN (HUMAN);.	Z50753 H.sapiens mRNA for GCAP-II/uroguanylin precursor
M36634 gene 1 "Human vasoactive intestinal peptide (VIP) mRNA, complete cds."	M83670 "Human carbonic anhydrase IV mRNA, complete cds"
T51571 3’ UTR 1 72250 P24480 CALGIZZARIN.	T52362 yb23g02.s1 Homo sapiens cDNA clone 72050 3’
M91463 gene 1 "Human glucose transporter (GLUT4) gene, complete cds."	H57136 yr08c08.s1 Homo sapiens cDNA clone 204686 3’ similar to SP:A40533 A40533 CAMP-DEPENDENT PROTEIN KINASE MAJOR MEMBRANE SUBSTRATE PRECURSOR—;
T62947 3’ UTR 2a 79366 60S RIBOSOMAL PROTEIN L24 (Arabidopsis thaliana)	U17077 "Human BENE mRNA, partial cds"
R97912 3’ UTR 2a 200181 SERINE/THREONINE-PROTEIN KINASE IPL1 (Saccharomyces cerevisiae)	T67077 ya52f06.s1 Homo sapiens cDNA clone 66563 3’ similar to SP:A40533 A40533 CAMP-DEPENDENT PROTEIN KINASE MAJOR MEMBRANE SUBSTRATE PRECURSOR—;
L41559 gene 1 "Homo sapiens pterin-4a-carbinolamine dehydratase (PCBD) mRNA, complete cds."	T55741 yb40d07.s1 Homo sapiens cDNA clone 73645 3’ similar to SP:TELO_RABIT P29294
R39209 3’ UTR 2a 23464 HUMAN IMMUNODEFICIENCY VIRUS TYPE I ENHANCER-BINDING PROTEIN 2 (Homo sapiens)	M12272 "Homo sapiens alcohol dehydrogenase class I gamma subunit (ADH3) mRNA, complete cds"
T90350 3’ UTR 2a 110964 MYOBLAST CELL SURFACE ANTIGEN 24.1D5 (Homo sapiens)	D63874 "Human mRNA for HMG-1, complete cds"
T54276 3’ UTR 1 69195 PROTEASOME COMPONENT C13 (HUMAN).	R71676 yj85e03.s1 Homo sapiens cDNA clone 155548 3’
R49459 3’ UTR 2a 38253 TRANSFERRIN RECEPTOR PROTEIN (Homo sapiens)	M26697 "Human nucleolar protein (B23) mRNA, complete cds"
Z24727 gene 1 "H.sapiens tropomyosin isoform mRNA, complete CDS."	M80244 "Human E16 mRNA, complete cds"
T51849 3’ UTR 2a 75009 TYROSINE-PROTEIN KINASE RECEPTOR ELK PRECURSOR (Rattus norvegicus)	L11708 "Human 17 beta hydroxysteroid dehydrogenase type 2 mRNA, complete cds"
K03460 gene 1 "Human alpha-tubulin isotype H2-alpha gene, last exon."	T46924 yb11b02.s1 Homo sapiens cDNA clone 70827 3’ similar to gb:U11863 AMILORIDE-SENSITIVE AMINE OXIDASE (HUMAN)
X61118 gene 1 Human TTG-2 mRNA for a cysteine rich protein with LIM motif.	U17899 "Human chloride channel regulatory protein mRNA, complete cds"
R06601 3’ UTR 2a 126458 METALLOTHIONEIN-II (Homo sapiens)	X73502 H. Sapiens mRNA for cytokeratin 20
	H09351 yl95g07.s1 Homo sapiens cDNA clone 46019 3’ similar to gb:D28480 MCM3 HOMOLOG (HUMAN);
	H06524 "yl78h01.s1 Homo sapiens cDNA clone 44386 3’ similar to gb:X04412 GELSOLIN PRECURSOR, PLASMA (HUMAN);"
	H77597 ys08a06.s1 Homo sapiens cDNA clone 214162 3’ similar to gb:X64177 H.sapiens mRNA for metallothionein (HUMAN);
	X15183 Human mRNA for 90-kDa heat-shock protein
	R50129 yj54h10.s1 Homo sapiens cDNA clone 152611 3’ similar to gb:J02939 4F2 CELL-SURFACE ANTIGEN HEAVY CHAIN (HUMAN);
	L03840 "Human fibroblast growth factor receptor 4 (FGFR4) mRNA, complete cds"
	T51261 yb03h03.s1 Homo sapiens cDNA clone 70133 3’
	H14506 ym18f10.s1 Homo sapiens cDNA clone 48421 3’
	H08393 yl92a10.s1 Homo sapiens cDNA clone 45395 3’
	T55200 yb43f08.s1 Homo sapiens cDNA clone 73959 3’ similar to gb:M10942_cds1 Human metallothionein-Ie gene (HUMAN)
	Z17227 H.sapiens mRNA for transmenbrane receptor protein
	H65066 yr69f12.s1 Homo sapiens cDNA clone 210575 3’ similar to SP:VIS1_RAT P28677 VISININ-LIKE PROTEIN 1; contains MER6 repetitive element;
	H17127 ym42e05.s1 Homo sapiens cDNA clone 50869 3’

### 6.2. Classification accuracy

**Fig 4:**
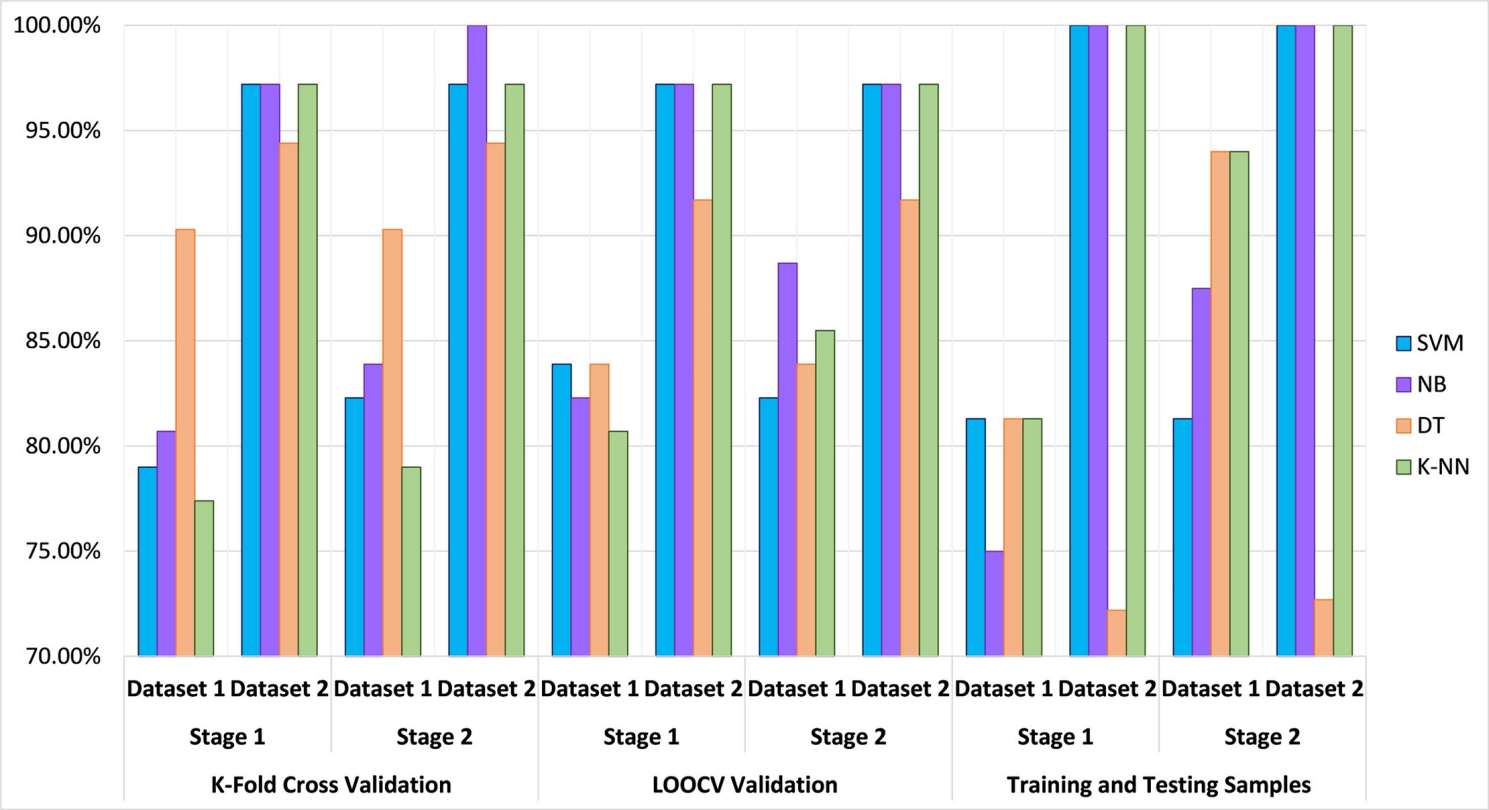
Evaluation of the proposed procedure’s classification accuracy using different testing models.

**Fig 5:**
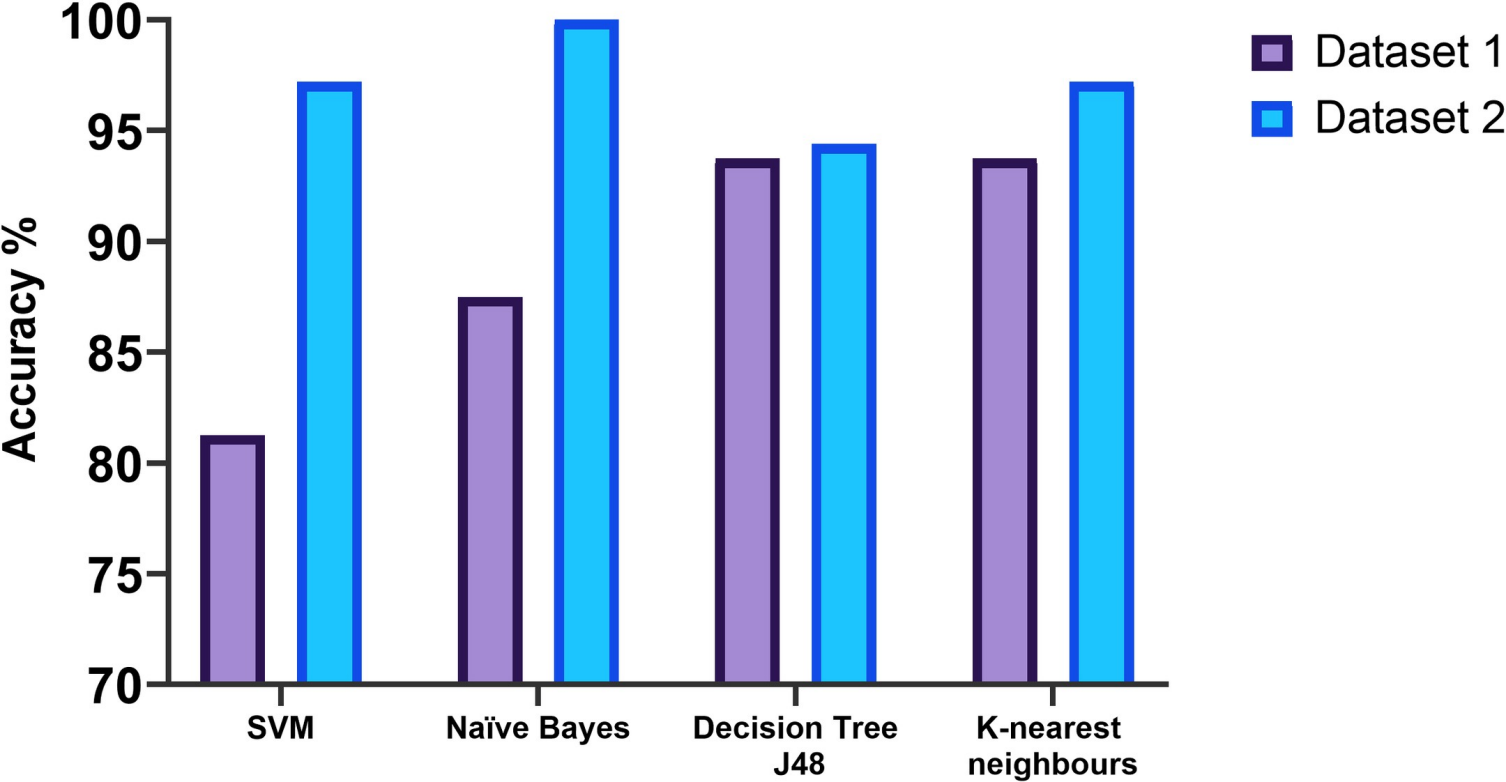
Evaluation of the proposed procedure’s classification accuracy using the best testing model with low error rates.

Table 5 compares the classification accuracy prediction results between stage 1 and stage 2, in order to verify the effectiveness of the proposed framework model with multiple testing models. From this table; it is recognized that the framework model has a clear direct effect on dataset 2, because of the data nature and the structure of the dataset. Since the dataset 2 had showed a very high classification accuracy in stage 1 (highest prediction accuracy 97% using K-fold and LOOCV), then the effect is slightly noticeable in stage 2 (highest prediction accuracy 100%). However, splitting out dataset 2 into training and testing validation sets will not have an effect on the dataset because of its nature and the smaller sample values included. While the effectiveness of the proposed framework model on dataset 1 is clearly noticed, as the highest accuracy in stage 1 is (90%),—in comparison with stage 2 that is (94%). Moreover, Fig 4 shows the evaluation results of the proposed procedure’s classification accuracy that was carried out using a number of algorithms: DT, K-NN, NB and SVM following the different testing models. In addition, Fig 5 renders the results that are considered as an appropriate process with lower predication error rates and less computational time when validating dataset 1 using the training and testing set, and for dataset 2 using the k-fold cross validation. The key findings as per Fig 5 are: 1) for the dataset 1 was that DT and K-NN performed best, with classification accuracy measured at (94%) when used as part of a two-stage process that began with a pre-selection stage. The least accurate algorithm was SVM (81.25%), whilst the level of performance achieved by NB (87.5%) was acceptable; 2) for the dataset 2 was that NB performed the best with a classification accuracy measured at (100%) under the implication of the two-stage model. The least accurate algorithm was DT (94.4%), whilst the level of performance achieved by both SVM and K-NN was (97.2%) see Table 5 and Fig 5.

**Table 5:** Comparison summary between stage one and stage 2 accuracy results.

**Classifier**	**K-Fold Cross Validation**			
	**Stage 1**		**Stage 2**	
	**Dataset 1**	**Dataset 2**	**Dataset 1**	**Dataset 2**
**SVM**	79.0%	97.2%	82.3%	97.2%
**NB**	80.7%	97.2%	83.9%	**100%**
**DT**	90.3%	94.4%	90.3%	94.4%
**K-NN**	77.4%	97.2%	79.0%	97.2%
**Classifier**	**LOOCV Validation**			
	**Stage 1**		**Stage 2**	
	**Dataset 1**	**Dataset 2**	**Dataset 1**	**Dataset 2**
**SVM**	83.9%	97.2%	82.3%	97.2%
**NB**	82.3%	97.2%	88.7%	97.2%
**DT**	83.9%	91.7%	83.9%	91.7%
**K-NN**	80.7%	97.2%	85.5%	97.2%
**Classifier**	**Training and Testing Samples**			
	**Stage 1**		**Stage 2**	
	**Dataset 1**	**Dataset 2**	**Dataset 1**	**Dataset 2**
**SVM**	81.3%	100%	81.3%	100%
**NB**	75.0%	100%	87.5%	100%
**DT**	81.3%	72.2%	**94.0%**	72.7%
**K-NN**	81.3%	100%	**94.0%**	100%

## 7. Analysis and discussion

**T**here is clear evidence to suggest that the hybrid multifilter method proposed here performs the task of feature selection better than similar approaches presented in the literature (see Table 1). Classification algorithms providing the best performance in classification were K-NN and DT (with an accuracy rate of 94%) for dataset 1, with NB emerging best algorithm for dataset 2, with an accuracy level of (100%) using the appropriate testing validation models as discussed in section 6.2.

A comparison of the proposed approach with those used in similar studies using the same dataset (the dataset 1) indicated that it achieved better, in terms of classification accuracy, than the method used by Zhang et al. [24] who resulted in (91.9%) accuracy using their proposed method of FSBRR and MI, followed by the K-NN. Also, our proposed model outperforms Abdi et al. [46], who reported a 90.32% level of accuracy when using mRMR and PSO, followed by SVM. The approach described in the current paper also out-performed that of Shutao et al. [48], who achieved an accuracy of 91.9% using a PSO+GA hybrid method, followed by SVM. Al Akadi et al. [52] reported a classification accuracy of 85.48%, using mRMR+GA, followed by SVM.

One difference with the previous studies is that they used fewer genes than the genes were selected for the present study; Abdi et al. [46] used 10.3 genes, reporting a classification accuracy that did not match that measured in the current study, whilst Shutao et al. [48] and Al Akadi et al. [52] used 18 and 40 genes, respectively. The classification accuracy achieved by [48] is (91.90%), while by [52] is (85.48%).

Another comparison was conducted with studies which used similar dataset (the dataset 2), and it was clearly indicated that our proposed method achieved better than Rathore et al. [8] who achieved an accuracy of (97.2%) while ours achieved (100%) which is also similar and better to approaches used by Al Snousy et al [80] who achieved also (97% - 100%). To confirm the comparative performance of the approach used in the current study, classification accuracy was 94% and 100%. The outcome of the research, therefore, is that, although some previous research was carried out using fewer genes, the approach described in this paper yielded better outcomes in terms of classification accuracy. This is because of the strategy to eliminate all bar the most informative and relevant genes.

It is noticed that most methods in the literature had achieved high classification accuracy when they applied the ML algorithms to a limited and selected number of genetic populations prior to classification not to all genes as in our case in which we didn’t exclude any gene from the beginning. It follows that our methods achieved an advanced level.

### 7.1. Methods employed for evaluation

Beside the training and testing samples, there are performance evaluation measures: (1) confusion matrix, (2) accuracy, (3) sensitivity, (4) specificity, (5) Matthews’s Correlation Coefficient (MCC), and (6) Receiver Operating Characteristic (ROC) Area.

A confusion matrix records True Positives (TP), which are the number of successfully identified positive samples, True Negatives (TN), which are the number of correctly identified negative samples, False Positives (FP), the samples erroneously diagnosed as being positive, and False Negatives (FN), those positive samples wrongly diagnosed as negative. An overall measure of classification efficiency is derived from this matrix, expressed as the percentage of correct diagnoses from the entire population of observations. According to Bolón-Canedo et al. [29], a sensitivity analysis measures the proportion of True Positives (TP), which, in practical terms, refers to the percentage of patients correctly diagnosed with cancer, whilst a specificity analysis refers to performance in identifying True Negatives (TN).

To achieve total predictive accuracy, an algorithm needs to perform with both 100% sensitivity and 100% specificity. The measurement of performance according to both of these indicators is known as a measure of ‘accuracy’, with the parameters TP, TN, FP and FN being used to calculate all of these measures.


$$\mathrm{Sensitivity}=\frac{TP}{TP+FN}$$ (2)

$$Specificity=\frac{TN}{TN+FP}$$ (3)

$$Accuracy=\frac{TN+TP}{TN+TP+FN+FP}$$ (4)

One test for the level of performance of an approach to solving binary problems is Matthews’ Correlation Coefficient (MCC), which yields values ranging from -1 to 1, where 1 describes a perfect classification performance and -1 indicates 100% error. An MCC value of zero is used to represent random prediction. The coefficient can be computed using the following equation:

$$MCC=\frac{TP\times TN-FP\times FN}{\sqrt{\left(\left(TP+FN\right)\left(TP+FP\right)\left(TN+FN\right)\left(TN+FP\right)\right)}}$$ (5)

An alternative evaluation method is the Receiver Operating Characteristic (ROC) Area. This method uses a two-dimensional graph to illustrate TP and FP outcomes in relation to defined thresholds. The observed rate of False Positives (FPR) is represented by the x-axis (sensitivity), whilst the y-axis shows the rate of True Positives (TPR) (1- specificity). The optimum plotted position on this graph is the coordinate (0, 1), which is called the ‘best classification’ or ‘perfect classification’, since it indicates both perfect sensitivity and perfect specificity.

### 7.2. Analysis of evaluation results

The confusion matrix used in the present study to assess the classification performance of the different approaches is shown in Tables 6 and 7. These data reveal that K-NN and DT performed best in classification of the sample of 62 genes using the dataset 1, while the NB performed the best in classification of 36 genes using the dataset 2. In dataset 1, these approaches accurately identified four positive, and eleven negative samples, with the sole error being the identification of a positive sample as being negative. However, in dataset 2, these approaches accurately identified 18 positive, and 18 negative samples.

**Table 6:** Confusion matrix of the present study for the evaluation of dataset 1.

Confusion Matrix	SVM		NB		DT		K-NN	
**Positive**	3	1	3	1	4	0	4	0
**Negative**	2	10	1	11	1	11	1	11
**TPR**	0.813		0.875		0.938		0.938	
**FPR**	0.229		0.208		0.021		0.021	

**Table 7:** Confusion matrix of the present study for the evaluation of dataset 2.

Confusion Matrix	SVM		NB		DT		K-NN	
**Positive**	18	0	18	0	18	0	18	0
**Negative**	1	17	0	18	2	16	1	17
**TPR**	0.972		1		0.944		0.972	
**FPR**	0.028		0		0.056		0.028	

**Fig 6:**
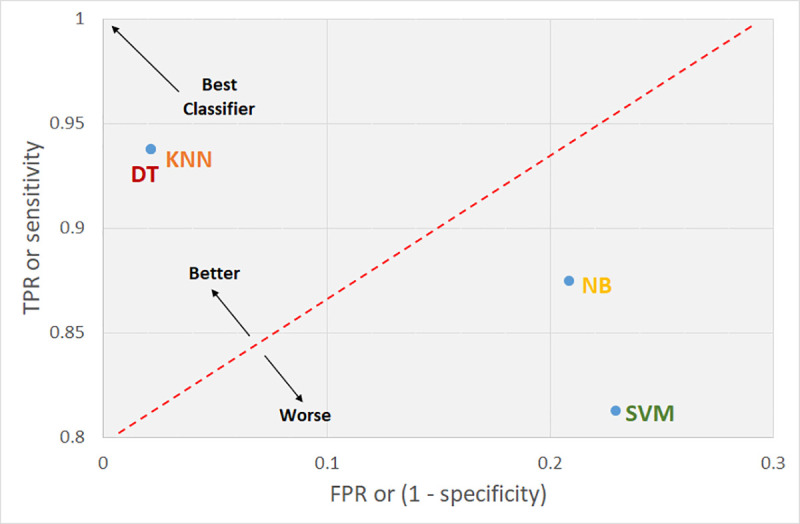
ROC curve of the present study for dataset 1.

**Fig 7:**
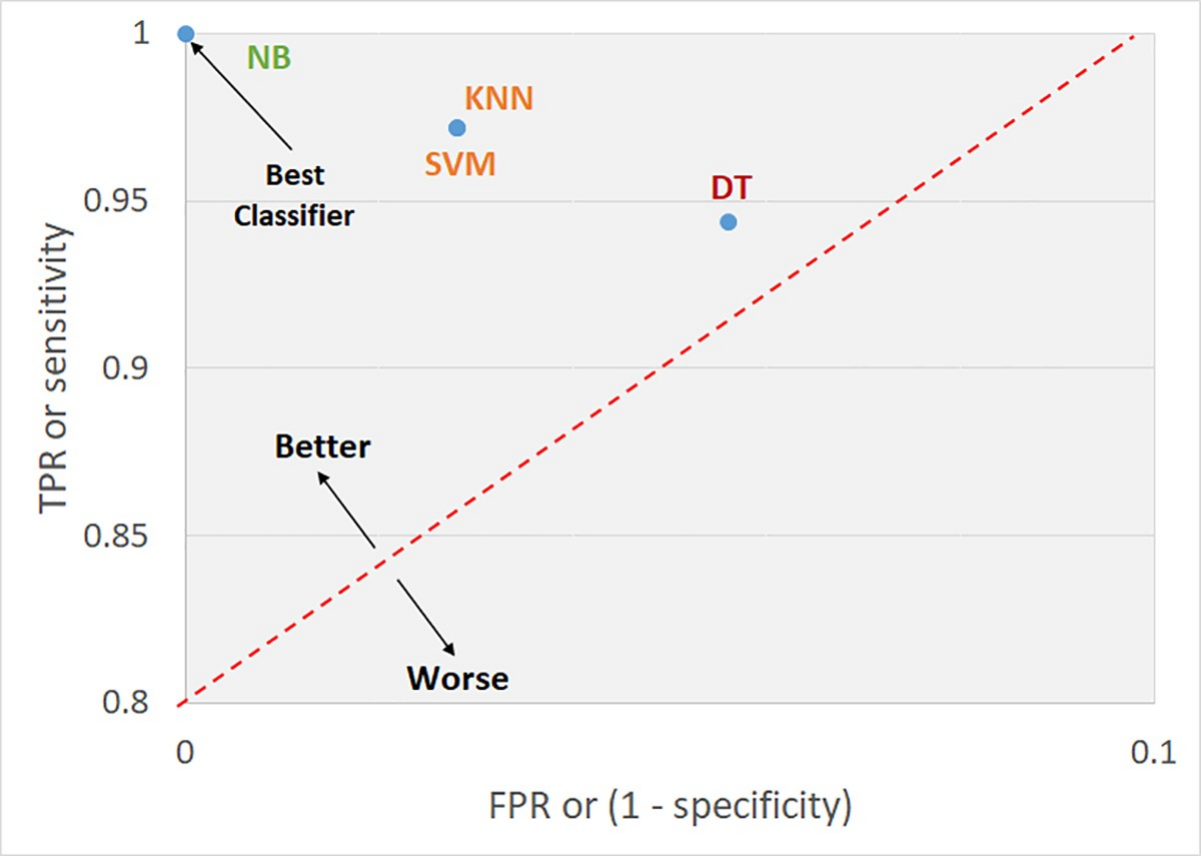
ROC curve of the present study for dataset 2.

The TPR and FPR data can be plotted graphically, as shown in Fig 6 for the dataset 1 and in Fig 7 for the dataset 2, with the TPR and FPR data being shown on the x-axis and y-axis, respectively (ROC Curve). The four approaches used are plotted in the two-dimensional space shown in Fig 6, which clearly indicates that DT and K-NN performed the best, with SVM yielding the worst performance for the dataset 1. On the other hand, Fig 7 clearly also indicates that NB performed the best dataset 2, with DT yielding the least but still considered reasonable.

The performance of the algorithms used in terms of accuracy, sensitivity, specificity and the Matthews’ Correlation Coefficient are shown in Table 8. Although the sample of genes eventually analysed was relatively small in both datasets, both specificity and sensitivity varied considerably, from 75% to 100%. In dataset 1, the equivalent figures for K-NN and DT were better, with both methods achieving 100% sensitivity and 91.7% specificity. In contrast, SVM performed poorly, with a sensitivity rate of 75% and an 83.3% level of specificity. In dataset 2, the figure of NB was the best with 100% sensitivity and specificity.

**Table 8:** Performance evaluation assessment for the experiment results.

Classifier	Dataset 1				Dataset 2			
	Accuracy	Sensitivity	Specificity	MCC	Accuracy	Sensitivity	Specificity	MCC
SVM	81.25	0.75	0.833	0.545	97.2	1	0.944	0.945
NB	87.50	0.75	0.917	0.667	100	1	1	1
DT	93.75	1	0.917	0.856	94.4	1	0.889	0.894
K-NN	93.75	1	0.917	0.856	97.2	1	0.944	0.945

In summary, the proposed model of the two stage multifilter outperforms other previously reported models in prediction accuracy and the numbers of genes selected within parentheses, evidenced in Table 9, for example, for dataset 1, it is 94.0% for 22 gens; for dataset 2, it is 100% for 35 gens. F-Score–Majority Voting [8] is doing very well with 95 gens to achieve 97% accuracy.

**Table 9:** Comparison of the proposed method with others reported in the literature using each dataset.

Method	Accuracy (%)
**Dataset 1**	
(FSBRR+MI)—K-NN [24]	91.90
(mRMR+PSO)—SVM [46]	90.32 (10)
(PSO+GA)—SVM [48]	91.90 (18)
(mRMR+GA)—SVM [52]	85.48 (40)
Filter (F-Score+IG)—Wrapper (SBE) + SVM [51]	87.50
(mRMR+GA)–SVM [50]	85.48
(PSO+GA)–DT [48]	85.50
(IG +GA)–GP [57]	85.48
(PCA) + GA–ANN [58]	83.33
**Our Proposed Model**	**94.0 (22)**
**Dataset 2**	
F-Score–Majority Voting [8]	97.22 (95)
Gain Ratio/ Chi-square + ensemble DT [80]	97.22
**Our Proposed Model**	**100 (35)**

An issue that might be investigated in the future, is the impact of different parameters on various algorithms’ level of classification performance. Also, the same method can be applied with other machine learning algorithms and can be extended to include other genetic datasets.

## 8. Conclusions and future work

The present study has proposed a two-stage hybrid multifilter data mining approach to feature selection, which has been shown to improve the diagnosis of colon cancer. The key improvement provided by the proposed approach was better classification of genes and accuracy of diagnosis. This was achieved through a decrease in the number of features considered in the analysis.

### 8.1. Achievements of the research

The proposed two-stage model delivered the following improvements:

Stage 1: The number of features used in the analysis was reduced by nearly 99% for both datasets included in this study, as compared with the sample population used at the beginning of the analysis. This was achieved through initially using the (IG+GA) selection approach.
Stage 2: During this stage, the approach reduced the number of genes used in the analysis to 22 for dataset 1 from an initial sample size of 2,000, and to 35 for dataset 2 from the initial sample size 6597. Furthermore, the amount of ‘noise’ in the data was lessened and genes having little or no relevance were eliminated. The approach also yielded enhanced levels of accuracy and displayed greater efficiency. The greatest classification accuracy was achieved by the K-NN and DT algorithms (at 94%) for dataset 1 and by NB algorithm (100%) for dataset 2.

The key outcome of the study is that the implementation of a feature selection procedure prior to the application of a classification algorithm provides more accurate predictions and diagnoses. The use of a hybrid multifilter process substantially reduced the number of features included in the dataset.

### 8.2. Future research

There are number of challenges for the further research to take on. Currently, we are going to focus on the investigations into:

1. the identifications of new variables in colon cancers.
2. the impact of different parameters on various algorithms’ level of classification performance.
3. ML methods for other complex genetic datasets in colon cancer case.
